# Hsa_Circ_0098181 Suppresses Hepatocellular Carcinoma by Sponging miR-18a-3p and Targeting PPARA

**DOI:** 10.3389/fphar.2022.819735

**Published:** 2022-02-21

**Authors:** Yuan-Yuan Luo, Ke-Gong Tao, Yi-Ting Lu, Bin-Bin Li, Kai-Ming Wu, Chen-Hong Ding, Fang-Zhi Yan, Yue Liu, Yong Lin, Xin Zhang, Xin Zeng

**Affiliations:** ^1^ Department of Gastroenterology, Shanghai East Hospital, Tongji University School of Medicine, Shanghai, China; ^2^ Department of Pathology, Shanghai Changzheng Hospital, Navy Military Medical University, Shanghai, China; ^3^ Department of Gastroenterology, Shanghai Changzheng Hospital, Navy Military Medical University, Shanghai, China

**Keywords:** hsa_circ_0098181, hepatocellular carcinoma (HCC), mir-18a-3p, PPARA, therapeutic target

## Abstract

Hepatocellular carcinoma (HCC) is one of the leading causes of cancer-related deaths, and its incidence is still high in China. This study aimed to investigate the circular RNAs (circRNAs) involved in the development of HCC and elucidate the mechanism. RNA sequencing found 72 downregulated circRNAs and 88 upregulated circRNAs in human HCC tissues, including hsa_circ_0098181, hsa_circ_0072309, hsa_circ_0000831, and hsa_circ_0000231. The reduction of hsa_circ_0098181 was confirmed in eight paired human HCC tissues, hepatoma cell lines, and CCL4/DEN-induced mouse HCC models by RT-qPCR. The FISH assay revealed that hsa_circ_0098181 is mainly located in the cytoplasm of hepatocytes in the paratumor tissues. Further log-rank analysis performed in 91 HCC patients demonstrated that low expression of hsa_circ_0098181 was related to poor prognosis. The plasmid and lentivirus overexpressing hsa_circ_0098181 were delivered into HCC cell lines. After hsa_circ_0098181 was upregulated, the proliferation, invasion, migration, and colony formation of HCC cell lines were inhibited, and the apoptosis was promoted. Moreover, exogenous hsa_circ_0098181 delivery mitigated the tumor formation ability of Huh7 in Balb/C nude mice. The dual-luciferase reporter assay and the RIP assay verified that hsa_circ_0098181 sponged miR-18a-3p to regulate PPARA. In addition, a rescue experiment found miR-18a-3p mimic partly reversed the suppression of hsa_circ_0098181 on proliferation, invasion, and migration of HCC cell lines. In conclusion, hsa_circ_0098181 can repress the development of HCC through sponging miR-18a-3p and promoting the expression of PPARA *in vitro* and *in vivo*, and hsa_circ_0098181 might be a therapeutic target for HCC.

## Introduction

Primary liver cancer is one of the leading causes of death from cancer (Akinyemiju et al*.*, 2017). At present, many risk factors, such as chronic hepatitis B, hepatitis C, alcohol, metabolic liver disease (especially nonalcoholic fatty liver), and chemical toxins, are involved in the development of liver cancer (Yang et al*.*, 2019). Pathologically, primary liver cancer can be divided into three types, including hepatocellular carcinoma (HCC, accounting for 75%), cholangiocarcinoma, and mixed types (Petrick et al., 2020). There is still no effective curative treatment for most patients, and surgical resection is only suitable for HCC patients in the early stage and without severe cirrhosis (El-Serag, 2011).

Circular RNA (circRNA) is a kind of non-coding RNA (ncRNA) which does not contain 5′ polarity and a 3′ polyadenylated tail, and has closed ring structures to ensure their stability (Chen and Yang, 2015). CircRNAs mainly exist in the cytoplasm or exosomes, and cannot be degraded by RNase R (Suzuki and Tsukahara, 2014). Since Thomas B. Hansen first proved that circRNAs can play regulatory roles as microRNA (miRNA) sponges in 2013, the sponge mechanism has become one of the main regulatory modes of circRNAs (Hansen et al., 2013; Li et al., 2020; Yang et al., 2020). In addition, circRNAs also regulate translation, bind with proteins, and affect gene transcription in recent studies (Prats et al., 2020; Zhou et al*.*, 2020).

Recently, accumulating evidence indicates that circRNAs can contribute to tumorigenesis, liver fibrosis, nonalcoholic fatty liver (NASH), liver regeneration, and other liver diseases (Wang G. et al., 2019; Ji et al*.*, 2020; Zhang and Wang, 2020; Zhao et al., 2020; Goodall and Wickramasinghe, 2021). Some research works believed that circRNAs might be promising biomarkers for the diagnosis and prognosis of HCC (Zhang and Wang, 2021). For instance, circRHOT1, a conserved and dramatically upregulated circRNA in HCC tissues, was found to promote the development and progression of HCC, and predict poor prognosis (Wang L. et al., 2019). However, the mechanism of circRNAs in HCC has not been fully elucidated to date.

It is well known that the adaptation of tumors to the local microenvironment is closely related to metabolic changes (Ray and Roy, 2018). As an important cell signaling and energy source, lipid metabolism is essential for cancer cells, which has been given more attention in liver cancer recently (Ray and Roy, 2018; Sangineto et al*.*, 2020). Peroxisome proliferator-activated receptor-α (PPARA), one of the core factors in the lipid metabolism, has been proven to participate in the development of tumor. The activation of PPARA resists angiogenesis and inflammation, reprograms glucose and lipid metabolism, and regulates the tumor microenvironment, and might have a protective effect on HCC (Lakshmi et al*.*, 2017; Zhang et al*.*, 2021).

In the current study, we identified the differentially expressed circRNAs in human HCC tissues by RNA sequencing. The expression of hsa_circ_0098181 was further detected in human HCC tissues, hepatoma cell lines, and mouse HCC models by RT-qPCR, and the impact of hsa_circ_0098181 on survival was analyzed in HCC patients. Moreover, we focused on investigating the effect of hsa_circ_0098181 on the development of HCC and illustrating the internal mechanism. Our results confirmed that increasing hsa_circ_0098181 mitigated the malignant phenotype of HCC *in vitro* and *in vivo*, and further revealed the downstream miRNA and its targets.

## Methods

### Hepatocellular Carcinoma Tissues and RNA Sequencing

The tumor and paratumor tissues were obtained from patients who underwent hepatectomy in Oriental Hepatobiliary Hospital and were pathologically diagnosed with HCC (Shanghai, China). RNA sequencing was performed using five paired human HCC tissues and their paratumor tissues to explore the circRNA signatures for HCC (Cloud Biotech, Shanghai, China). The study was approved by the Ethics Committee (Oriental Hepatobiliary Hospital, Shanghai, China; Shanghai East Hospital, Shanghai, China). Written informed consent was obtained from each patient.

### Cell Culture

Human hepatoma cell lines (Huh7 and Hep3B) were provided by the Type Culture Collection of the Chinese Academy of Sciences (Shanghai, China). Huh7 cells were cultured in Dulbecco’s modified Eagle’s medium (DMEM) containing 10% fetal bovine serum (FBS) and 1% penicillin–streptomycin. Hep3B cells were cultured in MEM with 10% FBS and 1% penicillin–streptomycin. All cells were cultured in a 5% CO_2_ humidified atmosphere at 37°C.

### Mice Hepatocellular Carcinoma Model

Primary HCC C57BL/6 mice (male) models were induced by N-nitrosodiethylamine (DEN) and carbon tetrachloride (CCL4). One dose of DEN (25 mg/kg) was injected intraperitoneally on the 14th day after birth. Carbon tetrachloride (CCL4) was administered intraperitoneally (2 ml/kg per week, CCL4: olive oil = 3:1) since the mice reached 10 weeks of age. Then the mice were sacrificed after 16 weeks. The liver of the mice was removed and collected for RNA extraction to further detect the level of hsa_circ_0098181.

### RNA Preparation, RNase R Treatment, and RT-PCR

Tissue and cell RNAs were isolated by TRIzol (TaKaRa, Tokyo, Japan). For RNase R treatment, 2 μg total RNA was incubated for 15 min at 37° with 6 U/μg RNase R (Epicentre Technologies, Madison, Wisconsin, United States) and mixed with PrimeScript RT Master Mix (TaKaRa, Tokyo, Japan) to synthesize cDNA. TB Green Premix EX Taq (TaKaRa, Tokyo, Japan) was applied for RT-qPCR. In addition, miRNAs’ reverse transcription and RT-qPCR were performed with the miDETECT A Track miRNA qRT-PCR Starter Kit (Ribobio, Guangzhou, China). The primers are listed in Table 1.

**TABLE 1:** Primers for the RT-qPCR.

Gene	Primer sequence (5′-3′)
hsa_circ_0098181	Forward: TGT​TGA​CAC​CTT​GAA​GCA​GAG
	Reverse: CATCTGCTTCCCCATACGGA
miR-18a-3p	RT-PCR: CCC​TAT​GTG​CTG​CTT​CTG​GAA​A
PPARA	Forward: TCAGTGTCGTCGAGTGCCTTCT
	Reverse: TTGTCGTTGCTGCTCTGTCCTG
hsa_circ_0072309	Forward: ACCGCTCAAATGTTATCTGGG
	Reverse: AATATCCATCATCTGTGCAATGC
hsa_circ_0000831	Forward: ACTTGGGGAACTGAGAAAGAAA
	Reverse: CCAAAATCCCACTGTTACCAAAT
hsa_circ_0000231	Forward: ACTAGCTTCTCCCAGGAACA
	Reverse: AGGAGATCTACACCAGTATCACA
GAPDH	Forward: TCTCTGCTCCTCCTGTTC
	Reverse: GTTGACTCCGACCTTCAC

### Fluorescence *In Situ* Hybridization

Fluorescence *in situ* hybridization (FISH) was used to explore the expression of hsa_circ_0098181 in human HCC tissues and determine its subcellular localization. The probe was provided by the RiboBio Company (Guangzhou, China). Each section was added with 20 μl pre-hybridizing solution in a wet box for 2–4 h and subsequently incubated with a 20-μM probe at 38–42°C overnight. Finally, the nuclei were stained with DAPI, and the sections were observed under a fluorescence microscope (OLMPUS BX51, Tokyo, Japan).

### Plasmid Transfection and Lentivirus Infection

The plasmid (PLC5-CIR carriers) carrying hsa_circ_0,098,181 or its control (Geneseed Biotech, Guangzhou, China) was transfected into hepatoma cell lines by Lipofectamine 2000 (Lipo 2000) and treated for 48 h. The lentivirus overexpressing hsa_circ_0098181 was constructed by Geneseed Biotech (Guangzhou, China). After virus infection, Huh7 cells were screened with Puro at a concentration of 2 mg/L, and a cell line stably expressing hsa_circ_0098181 was established in about 2–3 weeks. The cells were used to perform subcutaneous tumor formation and the rescue experiment.

### CCK-8 Assay

Cell proliferation was detected using CCK-8 (Cell Counting Kit-8; Dojinodo, Shanghai, China). About 3,000 cells (per plate) were incubated in 96-well plates. CCK-8 solution reagent (10 μl) was added to each well, and the cells were incubated at 37° for 1 h for five successive days. The absorbance at 450 nm was measured by a microplate daily (Bio Tek Synergy, Winooski, Vermont, United States).

### Transwell Assay

For the invasion assay, the upper chambers were coated with Mitrogel (Invitrogen, Carlsbad, CA, United States) at 37°C for 30 min. After adding trypsin into the plates, 10% of the FBS culture medium was used to stop digestion, and then the cells were washed with PBS twice. 500 μL of the culture medium with 10% FBS was put into the lower chamber of 24-well plates. Then 3 × 10^4^ cells were added into the upper chambers and incubated for 72 h. After dyeing with 0.1% crystal violet for 20 min, the invasion of cells was observed under a microscope. The migration of HCC cell lines was determined with a similar method, while the upper chambers did not have to be coated with Mitrogel. ImageJ software was used to quantify the degrees of invasion and migration of Huh7 and Hep3B cells.

### Apoptosis and Cell Cycle Assay

Apoptosis of cells was performed with AnnexinV-APC/7-AAD staining using the Apoptosis Detection Kit with 7-AAD (Biolegend, San Diego, CA, United States). The cell cycle was determined by the Cell Cycle Staining Kit (Multi Sciences, Hangzhou, China) according to the instructions. After staining, the apoptosis and cell cycle were detected by flow cytometry (BD Biosciences, San Jose, CA, United States).

### Tumor Formation Assay

Balb/C nude mice (male, six-week-old) were used to construct the xenograft model. Huh7 cells stably carrying hsa_circ_0098181 were subcutaneously injected into the backs of the nude mice (1 × 10^6^ cells per mouse). When the long diameter of the tumors reached about 2–3 mm, then the tumor volume was detected every 2 days. After 10 days, the mice were sacrificed. The stripped tumor tissues were used for subsequent RT-qPCR, Western blot assay, and immunohistochemistry staining.

### Bioinformatics Approaches for Target Gene Predicting

The potential downstream miRNAs of hsa_circ_0,098,181 were predicted using Circbank (www.circbank.cn) and Circinteratome (https://circinteractome.nia.nih.gov) websites. Similarly, the miRDB (www.mirdb.org) and miRmap (https://mirmap.ezlab.org) websites were used to determine the targets of miRNAs. Combined with the result of RNA sequencing, the target miRNA and mRNA were screened, and the binding sites were further predicted by Miranda v3.3a software.

### Western Blot Assay

A Western blot assay was carried out to detect the protein expression. The protein samples extracted with lysis buffer (with protease inhibitor) were loaded into the prepared SDS-PAGE gel and separated using gel electrophoresis. After the separated protein was transferred onto a PVDF membrane, the membrane was blocked with 5% milk for 1 h and incubated with the primary antibody (PPARA, BA1691, Boster Biological Technology, Pleasanton, CA, United States; β-actin, A5441, Sigma-Aldrich, St. Louis, MO, United States) overnight. Consequently, the secondary antibody was applied. One hour later, the membrane was scanned with the Odyssey infrared imaging scanner (LI-COR Odyssey system).

### Immunohistochemistry Staining

After dewaxing and hydration, the sections of mouse subcutaneous tumor tissues were treated with 3% hydrogen peroxide for 20 min and incubated with primary antibody (Ki-67, ab1667, Abcam, Cambridge, MA, United States; PPARA, BA1691, Boster Biological Technology, Pleasanton, CA, United States) overnight and then with secondary antibody for 60 min.

### Dual-Luciferase Reporter Gene Assay

To confirm the binding between circRNA and miRNA, the HEK-293 T cells were co-transfected with psiCHECK2-hsa_circ_0098181-WT/-MUT (Promega, Madison, WI, United States) and miR-18a-3p NC/mimic (Ribobio, Guangzhou, China). Similarly, psiCHECK2 PPARA-3′UTR-576bp-WT/MUT1/MUT2/MUT1+MUT2 (MUT1 and MUT2 were single mutations) and miR-18a-3p NC/mimic were co-transfected in HEK-293 T cells to verify the combination between miR-18a-3p and PPARA. The cells were quantified using a dual-luciferase reporter analysis kit (Promega, Madison, WI, United States).

### Radioimmunoprecipitation

The radioimmunoprecipitation (RIP) assay was used to verify the direct combination of hsa_circ_0098181 and Ago2, miR-18a-3p and Ago2 by the RNA Immunoprecipitation Kit (Geneseed Biotech, Guangzhou, China). First, 1 × 10^7^ Huh-7 cells were washed with PBS, and lysis buffer (containing protease inhibitor and RNase inhibitor) was added into the cells for 10 min. After centrifugation at 14000 g for 10 min, the supernatant was divided into three groups: input, Ago2, and IgG groups. 5 μg Ago2 (2897S, Cell Signaling Technology, Danvers, MA, United States) and IgG (3900S, Cell Signaling Technology, Danvers, MA, United States) antibodies were added separately into the Ago2 and IgG groups. After the binding of the antibody and the beads, the antigen was added. Finally, RNA was collected and reversed into cDNA for further RT-qPCR.

### Statistical Analysis

All data were analyzed by SPSS V.23.0 or GraphPad Prism 8 software. The results were analyzed by Student’s *t*-test and presented as the mean ± standard deviation (SD). The Kaplan–Meier method was used to analyze the survival in patients with different expression levels of hsa_circ_0098181. *p*-value < 0.05 was considered statistically significant (*p < 0.05, **p < 0.01, ***p < 0.001).

## Results

### Hsa_Circ_0098181 Decreases in Hepatocellular Carcinoma and Reduction of hsa_Circ_0098181 Is Related to the Poor Prognosis

**FIGURE 1:**
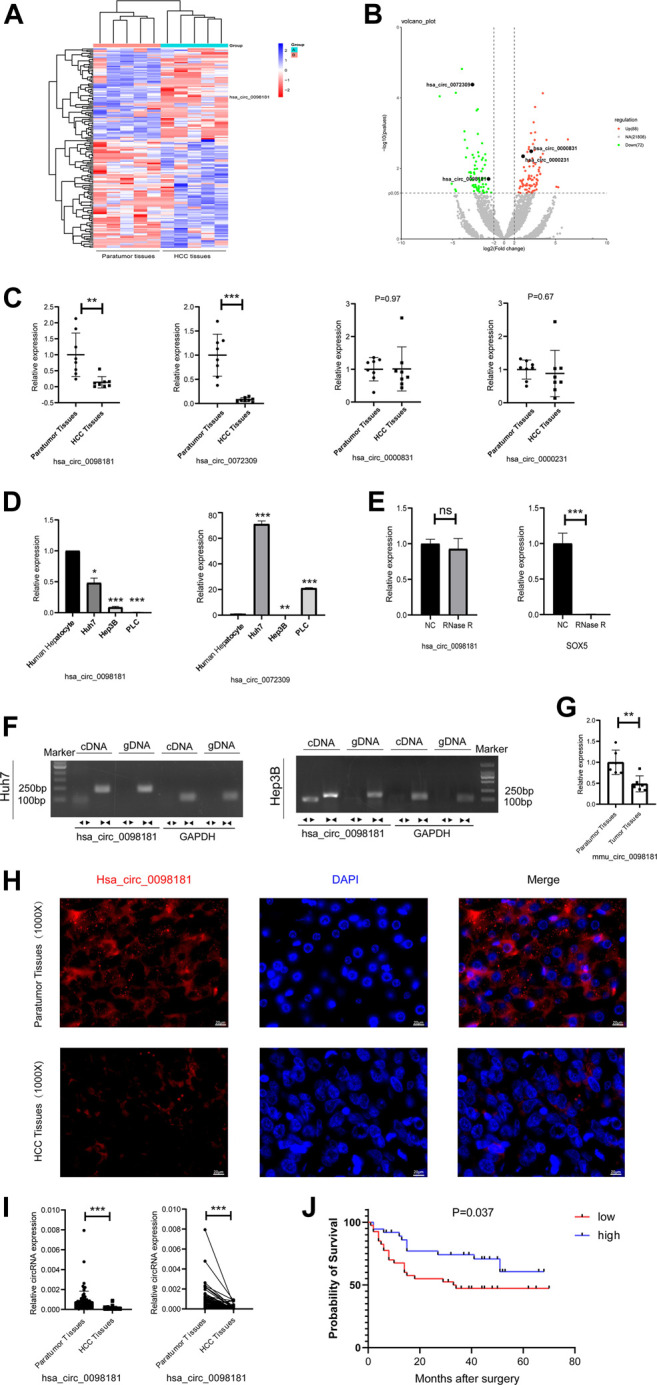
Hsa_circ_0098181 decreased in HCC and reduction of hsa_circ_0098181 was related to the poor prognosis. **(A,B)** Differentially expressed circRNAs in human HCC and paratumor tissues (heatmap and volcano map). **(C)** The RNA level of 4 circRNAs (hsa_circ_0098181, hsa_circ_0072309, hsa_circ_0000831, and hsa_circ_0000231) in paired human HCC tissues and their paratumor tissues (*n* = 8) by RT-qPCR. **(D)** The expressions of hsa_circ_0098181 and hsa_circ_0072309 in three hepatoma cell lines (Huh7, Hep3B, and PLC). **(E,F)** The RNase R resistance test and PCR amplification with convergent and divergent primers verified the ring structure of hsa_circ_0098181. **(G)** The reduction of circ_0098181 in the mouse HCC model induced by CCL4/DEN (*n* = 6). **(H)** FISH assay showed the enrichment of hsa_circ_0098181 (red) in liver paratumor tissues than that of HCC tissues (scale bar, 100 µm). **(I)** The expression of hsa_circ_0098181 was detected by RT-qPCR in 91 paired human HCC tissues. **(J)** Log-rank analysis showed low level of hsa_circ_0098181 was related to poor prognosis. Data were expressed as mean ± SD; *p < 0.05, **p < 0.01, ***p < 0.001.

RNA sequencing was performed to identify the differential circRNA profiles between human HCC tissues and their paratumor sections, and determine the circRNA signatures in HCC. It was shown that 72 circRNAs were less expressed and 88 circRNAs were up-regulated in human HCC tissues compared with their paratumor tissues (Figures 1A,B). Considering the length, nd homogeneous and differential expression of circular RNA (Zheng et al., 2016), 4 circRNAs (hsa_circ_0098181, hsa_circ_0072309, hsa_circ_0000831, and hsa_circ_0000231) at about 500 nt length were selected for further verification. In the paired human HCC tissues (*n* = 8), the levels of hsa_circ_0098,181 and hsa_circ_0072309 decreased obviously (Figure 1C). Moreover, the expressions of two circRNAs were quantified in three hepatoma cell lines (Huh7, Hep3B, and PLC). RT-qPCR indicated that hsa_circ_0098181 expression was reduced in all the liver cancer cell lines, compared with primary human hepatocytes (Figure 1D). According to RNA sequencing, the host gene of hsa_circ_0098181 was sex determining region Y-box protein 5 (SOX5), a transcriptional factor whose roles in hepatocellular carcinoma and other liver diseases were not clear. To verify the ring structure of hsa_circ_0098181, SOX5 was used as a control for linear RNA. With the RNase R treatment, hsa_circ_0098181 decreased slightly, while the transcription of SOX5 decreased significantly (Figure 1E). After divergent primer amplification, there were obvious bands in the cDNA in Huh7 and Hep3B cells (Figure 1F). These results demonstrated that hsa_circ_0098181 had a circular structure. Due to the high homology (92%) of circ_0098181 in humans and mice, we then tested the expression of circ_0098181 in the mice HCC model induced using CCL4/DEN. As expected, the reduction of circ_0098181 was verified in mouse HCC tissues (*n* = 6, *p* < 0.01, Figure 1G). The FISH probe revealed the enrichment of hsa_circ_0098181 in paratumor tissues compared with human HCC tissues. Additionally, the result also found hsa_circ_0098181 dominantly located in the cytoplasm of hepatocytes (Figure 1H). Furthermore, to clarify the role of hsa_circ_0098181 on HCC prognosis, we determined the expression of hsa_circ_0098181 in 91 paired human HCC tissues by RT-qPCR. Our observation showed that hsa_circ_0098181 expression was reduced in human HCC tissues compared with their paratumor tissues (Figure 1I, *p* < 0.001) and low expression of hsa_circ_0098181 was related to the poor prognosis (Figure 1J, *p* = 0.037). These results indicated the potential of hsa_circ_0098181 as a prognosis biomarker and therapeutic target for HCC.

### Hsa_Circ_0098181 Inhibits Malignant Phenotype of HCC *In Vitro* and *In Vivo*

**FIGURE 2:**
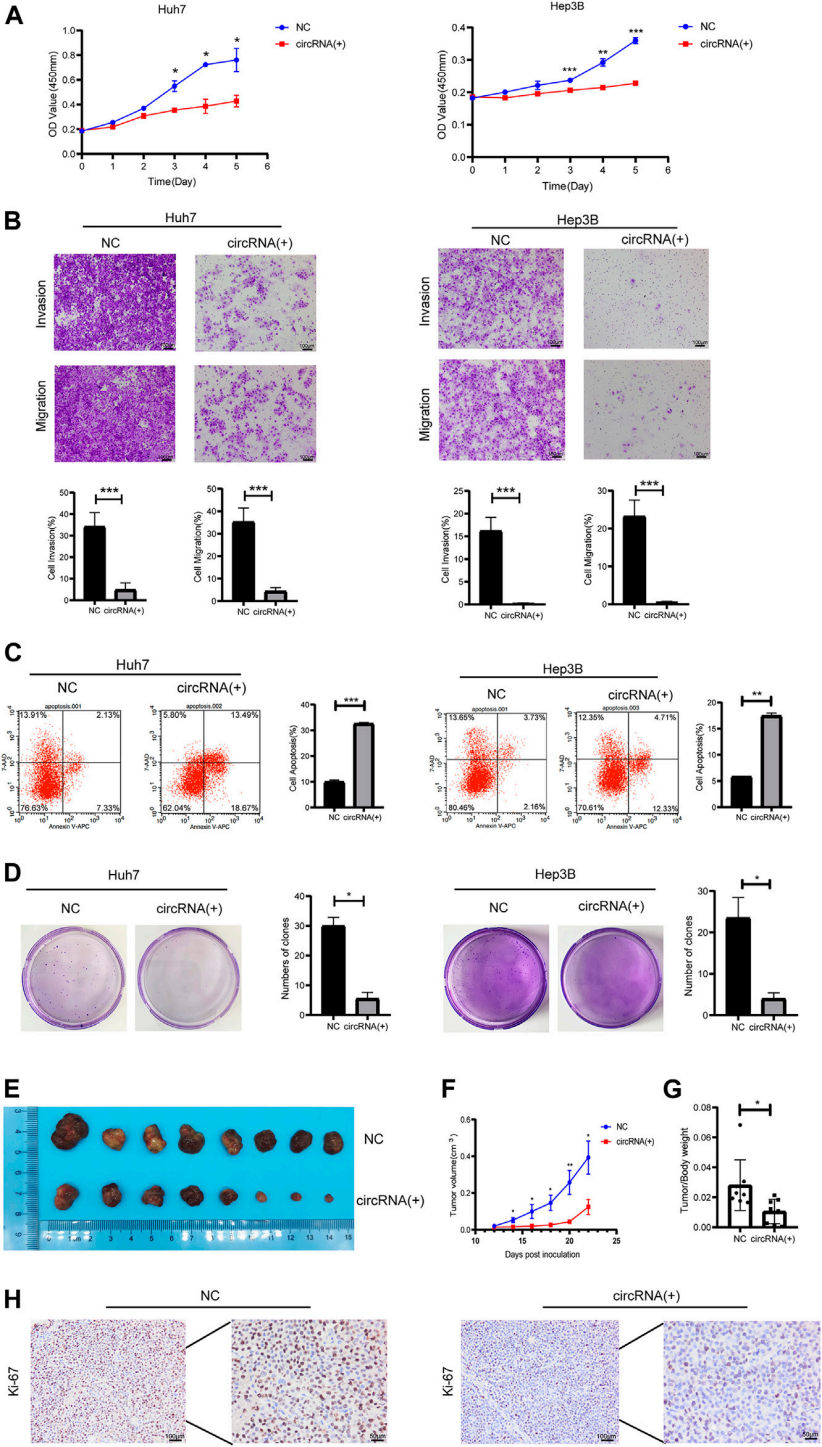
Hsa_circ_0098181 mitigated the malignant phenotype of HCC *in vitro* and *in vivo*. After exogenous hsa_circ_0,098,181 delivery, the proliferation **(A)**, invasion, migration **(B)**, and colony formation **(D)** were suppressed in both Huh7 and Hep3B. **(C)** The apoptosis was promoted by hsa_circ_0098181 in Huh7 and Hep3B in AnnexinV-APC/7-AAD staining. **(E)** Exogenous hsa_circ_0,098,181 delivery inhibited the subcutaneous tumor formation ability of Huh7. **(F)** Growth curve of xenograft tumors derived from stable Huh7 cells infected with lentivirus (*n* = 8). **(G)** The tumor weight/body ratio of the mice in hsa_circ_0098181 overexpression group was lower. **(H)** Immunohistochemical staining for Ki-67 in subcutaneous xenograft mice (scale bar, 100 µm). Data were expressed as mean ± SD; *p < 0.05, **p < 0.01, ***p < 0.001.

Plasmids carrying hsa_circ_0098181 significantly upregulated the expression of hsa_circ_0098181 in Huh7 and Hep3B cells (Supplementary Figure S1). After exogenous hsa_circ_0098181 delivery, the proliferation of HCC cells was suppressed whether in Huh7 or Hep3B cells (Figure 2A). Moreover, hsa_circ_0098181 significantly inhibited the invasion and migration of Huh7 and Hep3B cells (*p* < 0.001, Figure 2B). AnnexinV-APC/7-AAD staining showed that hsa_circ_0098181 aggravated the apoptosis rate in Huh7 (*p* < 0.001) and Hep3B cells (*p* < 0.01, Figure 2C). In addition, hsa_circ_0098181 up-regulation had little effect on the cell cycle (Supplementary Figure S2). Colony formation assays suggested that hsa_circ_0,098,181 alleviated the colony formation of HCC cells (*p* < 0.001, Figure 2D). Furthermore, Huh7 cells infected with lentivirus carrying hsa_circ_0098181 were used to construct a xenograft mouse model. As expected, hsa_circ_0098181 apparently mitigated the tumor size (Figures 2E, F) and the tumor weight/body weight ratio (Figure 2G). Immunohistochemistry staining revealed that Ki-67 decreased significantly in the group with overexpression of hsa_circ_0098181 (Figure 2H). All the above observations inferred that hsa_circ_0098181 was capable of alleviating the malignant phenotype of HCC *in vitro* and *in vivo*.

### Hsa_Circ_0098181 Sponges miR-18a-3p to Regulate PPARA

**FIGURE 3:**
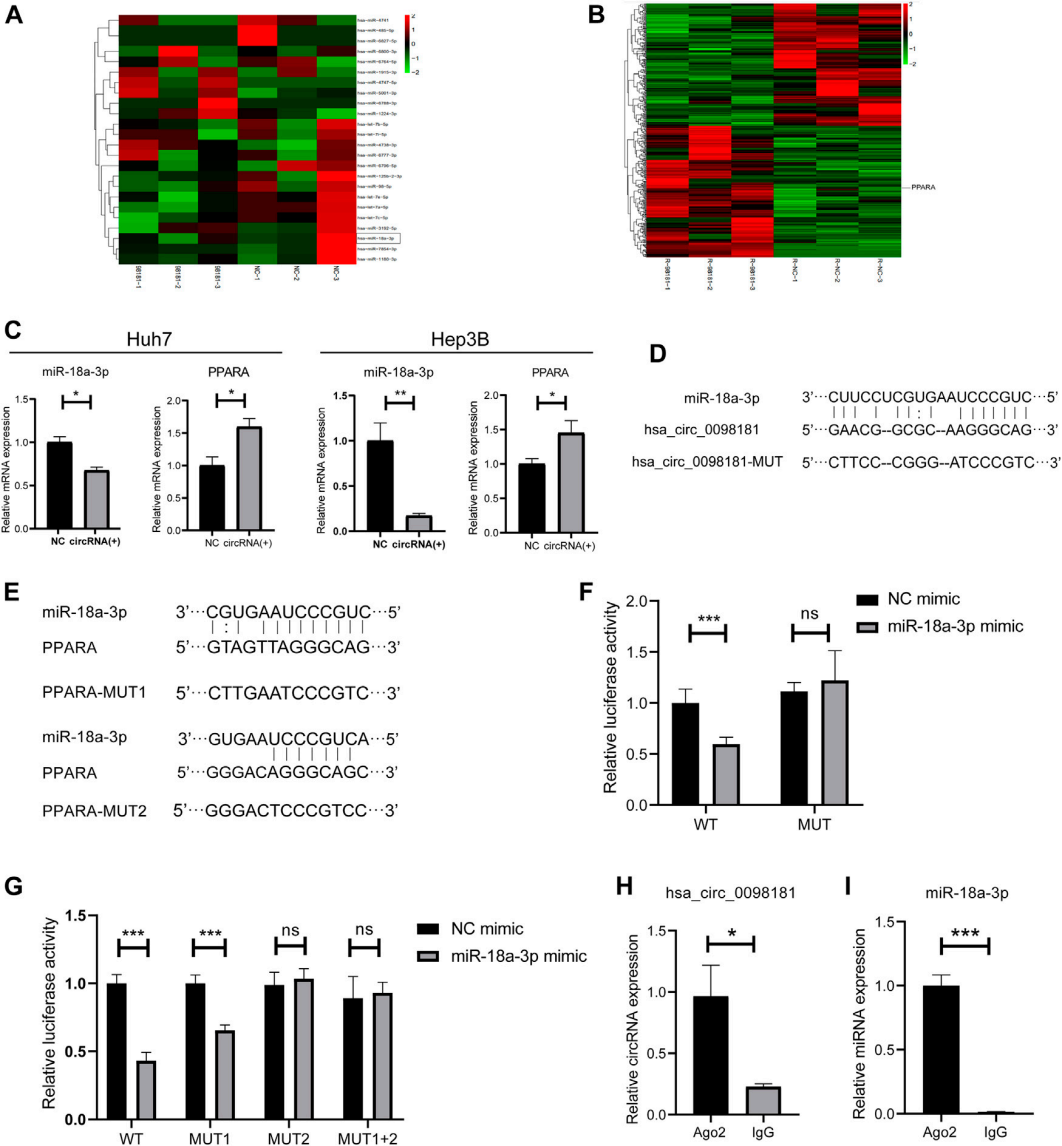
Hsa_circ_0098181 sponged miR-18a-3p to regulate PPARA. **(A)** The miRNA sequencing of hsa_circ_0098181 overexpressed Huh7 cells and bioinformatics analysis indicated that miR-18a-3p sponged hsa_circ_0098181. **(B)** The mRNA sequencing of hsa_circ_0098181 overexpressed Huh7 cells and bioinformatics analysis suggested PPARA as the direct target of miR-18a-3p. **(C)** RT-qPCR showed that hsa_circ_0098181 significantly decreased the RNA level of miR-18a-3p and increased PPARA transcription in the Huh7 and Hep3B. **(D,E)** Miranda v3.3a software predicted the binding sites of miR-18a-3p and the target region of 3′UTR of hsa_circ_0098181 and PPARA. The mutation sequence of the 3′UTR region of hsa_circ_0098181 and PPARA were also revealed. **(F,G)** Dual-luciferase reporter assay elucidated the combination of miR-18a-3p and the 3′UTR region of hsa_circ_0098181 and PPARA. **(H,I)** RIP experiment demonstrated both hsa_circ_0098181 and miR-18a-3p combined Ago2. Data were expressed as mean ± SD; *p < 0.05, **p < 0.01, ***p < 0.001.

It is well documented that circRNAs are often regarded as miRNA sponges, which combine with miRNA to affect downstream target genes (Liu et al., 2019; Su et al*.*, 2019; Yang et al., 2021). Based on the RNA sequencing in the hsa_circ_0098181 overexpressed Huh7 cells and bioinformatics analysis for target gene predicting, miR-18a-3p was considered as the potential target of hsa_circ_0098181 (Figure 3A). Similarly, PPARA was determined to be the downstream of miR-18a-3p (Figure 3B). Further confirming the modification of hsa_circ_0098181 on its target signaling, hsa_circ_0098181 was upregulated in Huh7 and Hep3B. RT-qPCR suggested that hsa_circ_0098181 significantly decreased the RNA level of miR-18a-3p and increased PPARA transcription in both cell lines (Figure 3C). Binding site prediction by Miranda v3.3a software revealed one binding site between hsa_circ_0098181 and miR-18a-3p, and two binding sites between miR-18a-3p and PPARA. Subsequently, a dual-luciferase reporter assay elucidated the combination of miR-18a-3p and the 3‘ UTR region of hsa_circ_009881 and the direct binding of miR-18a-3p and PPARA (*p* < 0.001, Figures 3D–G). RIP experiments revealed that hsa_circ_0098181 and miR-18a-3p combined with Ago2 directly (Figures 3H,I). These findings indicated that miR-18a-3p was the target of hsa_circ_0,098,181 and that miR-18a-3p interacted with PPARA directly.

### Hsa_Circ_0098181 Mitigates the Development of Hepatocellular Carcinoma *via* Sponging miR-18a-3p and Targeting PPARA

**FIGURE 4:**
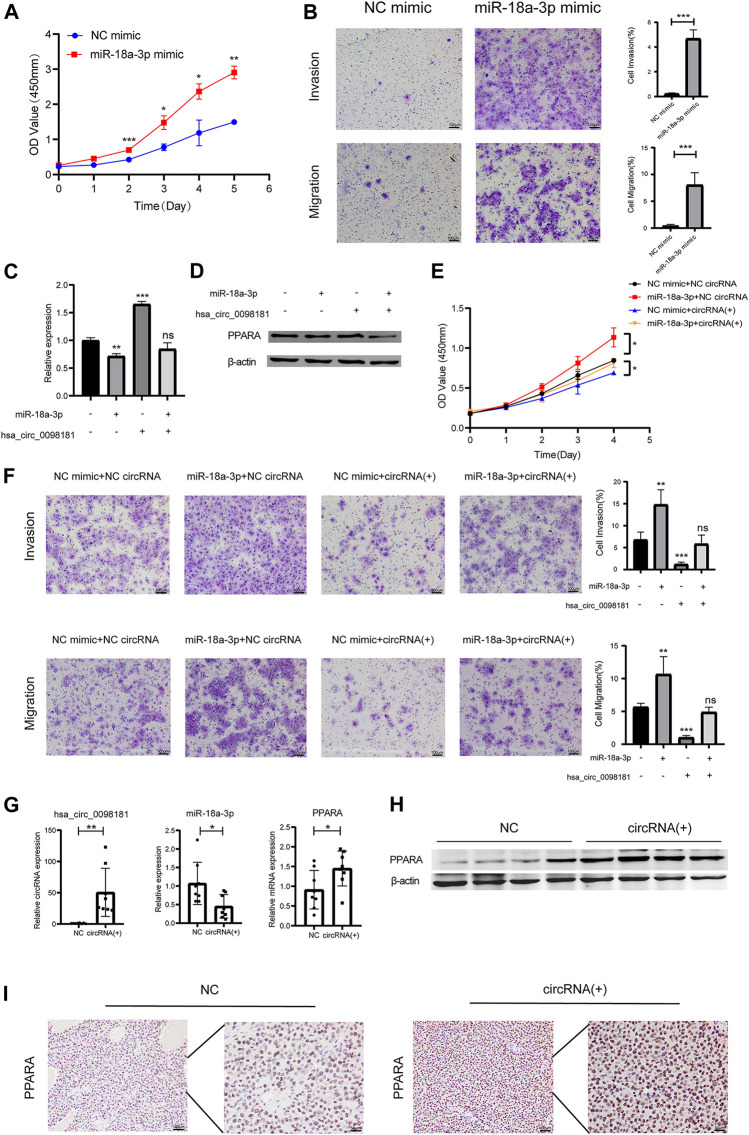
Hsa_circ_0,098,181/miR-18a-3p/PPARA. **(A)** CCK-8 assay showed the proliferation of Huh7 cells after miR-18a-3p mimic/NC mimic transfection. **(B)** Transwell experiment revealed that miR-18a-3p mimic repressed the invasion and migration of Huh7 cells. **(C,D)** The rescue experiment indicated that miR-18a-3p mimic partially decreased the RNA and protein level of PPARA induced by hsa_circ_0098181. **(E,F)** CCK-8 and Transwell assays suggested that miR-18a-3p mimic reversed the suppression of hsa_circ_0098181 on the proliferation, invasion, and migration of Huh7 cells. **(G,I)** RT-qPCR, Western blot, and immunohistochemical staining detected the expression of hsa_circ_0098181, miR-18a-3p, and PPARA in xenograft tumors (scale bar, 100 µm). Data were expressed as mean ± SD; *p < 0.05, **p < 0.01, ***p < 0.001.

According to Wang’s research (Wang L. et al., 2021), miR-18a-3p levels increased in HCC tissues. However, the mechanism of miR-18a-3p in liver cancer still remains elusive. Therefore, we transfected miR-18a-3p mimic into Huh7 cells and investigated the effect of miR-18a-3p on the biological behavior of HCC cells. The results showed that upregulating miR-18a-3p obviously aggravated the proliferation, invasion, and migration of Huh7 (Figures 4A, B). To further explore the effect of hsa_circ_0098181 on miR-18a-3p/PPARA, we conducted a rescue experiment. After co-administration of lentivirus carrying hsa_circ_0098181 and miR-18a-3p/NC mimic, the increase of the RNA and protein expression of PPARA induced by hsa_circ_0098181 could be impaired by miR-18a-3p mimic (Figures 4C, D). Moreover, miR-18a-3p mimic also partially reversed the suppression of hsa_circ_0098181 on the proliferation, invasion, and migration of Huh7 cells (Figures 4E, F). In the tumor formation assay, the xenograft tissues derived from Huh7 cells carrying hsa_circ_0098181 showed a higher RNA level of hsa_circ_0,098,181 than those controls. In parallel, hsa_circ_0098181 also reduced miR-18a-3p levels (*p* < 0.05) and promoted the transcription of PPARA in xenograft tissues (*p* < 0.05, Figure 4G). Additionally, Western blot and immunohistochemical staining found the expression of PPARA increased significantly in hsa_circ_0098181 overexpressed tissue sections (Figures 4H, I).

## Discussion

Hepatocellular carcinoma (HCC) is a leading cause of cancer-related death in humans with a poor prognosis, and the median survival time is about 21 months (Tabrizian et al*.*, 2015; Akinyemiju et al*.*, 2017). Therefore, it is still meaningful to find the potential target genes for the treatment of HCC. CircRNAs mainly exist in the cytoplasm or exosomes and have resistance to RNase R with conserved and stable ring structures (Xu T. et al., 2017). In recent years, many studies have focused on the role of circRNAs on HCC (Yu et al*.*, 2018; Huang et al., 2020). Nevertheless, the mechanism of most circRNAs in HCC has not been well documented, and there is no research about hsa_circ_0098181 in liver cancer. In this report, we found that hsa_circ_0098181 was down-regulated in human HCC tissues, and overexpression of hsa_circ_0098181 alleviated the malignant phenotype of HCC both *in vitro* and *in vivo*.

Previous research works have screened out several circRNAs whose levels varied in HCC tissues, including ciRS-7, hsa_circ_0005075, hsa_circ_0005986, and hsa_circ_0004018 (Capel et al., 1993; Xu L. et al., 2017; Lei et al., 2019). Among them, ciRS-7 and hsa_circ_0005075 were upregulated, while hsa_circ_0005986 and hsa_circ_0004018 were downregulated in HCC. It has been reported that circRNA profiles were related to HCC tumor size, serum AFP, and survival, suggesting circRNAs as biomarkers for HCC diagnosis and prognosis. Our findings revealed the differentially decreased expression of hsa_circ_0098181 in HCC tissues, hepatoma cell lines, and animal HCC models, and confirmed the association between hsa_circ_0098181 and prognosis, indicating that hsa_circ_0098181 might be a novel potential biomarker for HCC diagnosis and prognosis prediction. To our knowledge, this is the first time to investigate the role of hsa_circ_0098181 in HCC.

It is generally believed that the proliferation, invasion, migration, and colony formation abilities of tumor cells were related to the malignant phenotype of HCC. In this study, overexpression of hsa_circ_0098181 obviously inhibited the proliferation, invasion, migration, and colony formation of Huh7 and Hep3B cells, and the tumorigenicity of HCC cell lines was remarkedly repressed after hsa_circ_0098181 delivery. This observation suggested the prominent antitumor effect of hsa_circ_0098181 on HCC.

CircRNAs can often act as miRNA sponges to regulate the downstream genes. Our luciferase reporter assay and RIP assay verified the direct interaction between hsa_circ_0098181 and miR-18a-3p. Enrichment of miR-18a-3p has been found in liver cancer (Wang L. et al., 2021), but studies about its mechanism in HCC are limited. Previous studies have documented the promoting effect of miR-18a-3p on gastric and breast cancer. In *H. pylori*-associated gastric cancer, miR-18a-3p was increased and upregulating miR-18a-3p stimulated the growth and motility of gastric cancer cell lines *in vitro* (Tsai et al., 2020). In addition, miR-18a-3p played a critical role in the inhibitory effect of homoharringtonine on breast cancer *via* targeting the AKT-mTOR pathway, and miR-18a-3p inhibitor facilitated HHT-induced cancer cell apoptosis (Wang LB. et al., 2021). Though the mechanism of miR-18a-3p in HCC remains unclear, according to the research works in other tumors and the obvious discrepancy of miR-18a-3p expression between HCC and normal liver tissues/cells, we believe that miR-18a-3p may participate in the development of liver cancer. As expected, in our study, miR-18a-3p mimic aggravated the proliferation, invasion, and migration. This is also the first time to elucidate the effect of miR-18a-3p on HCC.

PPARA is a nuclear transcription factor enriched in the human liver and is capable of inhibiting fatty acid oxidation (FAO) and reducing lipid deposition. As the core regulator of the lipid metabolism, PPARA can be activated by fatty acids and has the ability to suppress inflammation (Kersten and Stienstra, 2017). Nowadays, PPARA is regarded as a vital target for non-alcoholic steatohepatitis (NASH) and liver fibrosis. Elafibranor, a PPARA agonist, has been proven to induce resolution of NASH without fibrosis worsening in the clinical study (Ratziu et al., 2016). In this study, histological damage (hepatocyte ballooning, non-alcoholic fatty liver disease activity score (NAS), lobular inflammation, and steatosis), liver enzymes, lipids and glucose profiles, and markers of systemic inflammation of patients with NASH were all significantly reduced after treatment with elafibranor at a dose of 120 mg/d for 1 year. In 2017, IVA337, another new PPAR (including PPARA) agonist, revealed its ability to suppress the proliferation and activation of human hepatic stellate cells (HSCs), and alleviate mouse liver fibrosis induced by CCL4 (Wettstein et al., 2017). These studies encourage the application of the PPARA agonists for the therapy of various liver diseases.

Considering the essential role of inflammation and lipid metabolism in HCC, PPARA is believed to be a potential anti-HCC target (Alannan et al., 2020). Recently, PPARA was found to be significantly down-regulated in human liver tumor tissues (Drakaki et al., 2015) and played a vital part in the effect of many non-coding RNAs on HCC. For instance, PPARA was a direct target of miR-9-5p and was critical for the function of LINC00467 in HCC (Cai et al., 2019). In addition, miR-580-5p can be combined with PPARA in HCC cells to stimulate the production and secretion of C-C chemokine ligand 2 (CCL2) and modify the tumor microenvironment (Wang X. et al., 2021). However, there is no clinical report on the therapeutic effect of PPARA agonists on HCC to date. In the present study, RNA sequencing, bioinformatics, and dual-luciferase reporter assay all indicated that PPARA was the direct target of miR-18a-3p and exogenous hsa_circ_0098181 administration modified the level of miR-18a-3p and enhanced PPARA expression. Thus, we believed hsa_circ_0098181 exerted antitumor effects *via* inhibiting miR-18a-3p and promoting PPARA expression. The rescue experiments confirmed that miR-18a-3p mimic partially reversed the antitumor effect of hsa_circ_0098181 on HCC and its impact on PPARA expression. This finding further clarified the regulation of hsa_circ_0,098,181 on miR-18a-3p and PPARA.

Taken together, our current study demonstrated the reduction of the hsa_circ_0098181 level in human HCC and revealed the repression of hsa_circ_0098181 on the malignant phenotype of HCC *in vitro* and *in vivo*. Moreover, the further study found that hsa_circ_0098181 played anti-HCC effect *via* sponging miR-18a-3p and targeting PPARA. Thus, hsa_circ_0098181 might be a promising biomarker and therapeutic target for liver cancer.

## Data Availability

The datasets presented in this study can be found in online repositories. The names of the repository/repositories and accession number(s) can be found below: https://www.ncbi.nlm.nih.gov/geo/, GSE190174; GSE190254; GSE190255.

## Abbreviations

CCL4, carbon tetrachloride; CircRNAs, circular RNAs; DEN, n-nitrosodiethylamine; DMEM, Dulbecco’s modified Eagle’s medium; FBS, fetal bovine serum; FISH, fluorescence *in situ* hybridization; HCC, hepatocellular carcinoma; MiRNAs, micro-RNAs; NASH, non-alcoholic fatty liver; NcRNA, non-coding RNA; PPARA, peroxisome proliferator-activated receptor-α.
