# Supplementary material for: Hsa_Circ_0098181 Suppresses Hepatocellular Carcinoma by Sponging miR-18a-3p and Targeting PPARA
Source: Front Pharmacol. 2022 Feb 21;13:819735. doi: 10.3389/fphar.2022.819735 (PMC8899401; doi:10.3389/fphar.2022.819735)

**Supplementary figures**

Supplementary figure 1. Transfection efficiency of plasmids

The transfection efficiency of Huh7 (A) and Hep3B (B) cells after transfecting 48 h with plasmids. Data were expressed as mean ± SD; ****p* < 0.001.


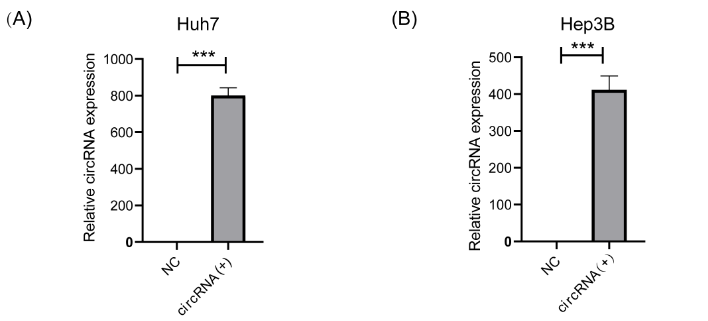


Supplementary figure 2. Cell cycle analysis of hsa_circ_0098181 in Huh7 and Hep3B

Cell cycle analysis was performed with hsa_circ_0098181 and control plasmids and the cell cycle distributions were displayed. Increasing hsa_circ_0098181 had little effect on cell cycle.


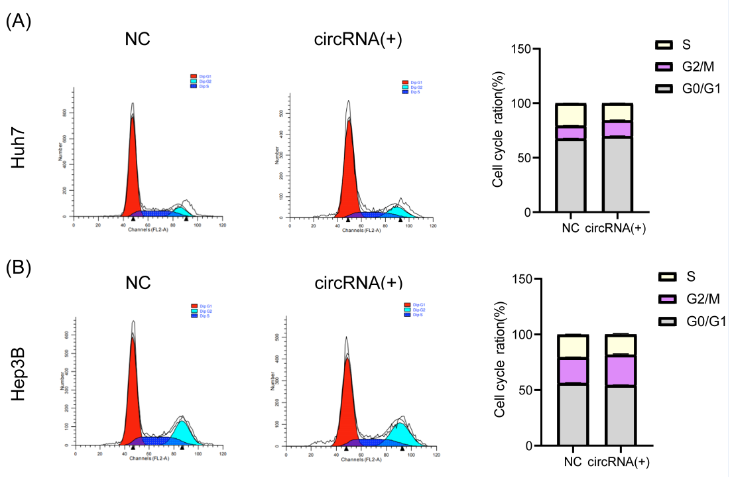

Supplement: Supplementary file 1 [file DataSheet1.DOCX]
